# A Rare Case of Multilocular Mesothelial Inclusion Cysts of the Pericardium: Diagnosis, Treatment, Follow Up, with Comprehensive Review of the Literature

**DOI:** 10.3390/jpm15110529

**Published:** 2025-11-02

**Authors:** Ali Shadmanian, Kosha Patel, Endre Alács, Henriette Gavallér, Szilva Agocs, Miklós Bitay

**Affiliations:** 1 Department of Internal Medicine, Cardiology Center, Department of Cardiac Surgery, Albert Szent-Györgyi Clinical Centre, University of Szeged, 6725 Szeged, Hungary; shadmanian.ali@med.u-szeged.hu (A.S.); patel.kosha@o365.u-szeged.hu (K.P.); 2 Department of Anesthesiology and Intensive Therapy, University of Szeged, 6725 Szeged, Hungary; alacs.endre@med.u-szeged.hu (E.A.); balintne.agocs.szilvia@med.u-szeged.hu (S.A.); 3 Department of Internal Medicine, Cardiology Centre, Acute Internal Medicine Department, University of Szeged, 6725 Szeged, Hungary; gavaller.edit.henriette@med.u-szeged.hu

**Keywords:** pericardial cyst, benign multicystic mesothelioma, mesothelial inclusion cyst, pericardiectomy, cardiac tumor, recurrence

## Abstract

Background: Multilocular mesothelial inclusion cysts—also known as benign multicystic mesothelioma (BMM)—are rare, typically arising in the peritoneal cavity. Pericardial involvement is extremely uncommon and can pose diagnostic and therapeutic challenges due to their recurrent and infiltrative nature. Accurate diagnosis and surgical strategy are critical for management and recurrence prevention. Methods: We present the case of a 36-year-old woman with a prior history of malignant melanoma who developed recurrent multilocular cystic masses of the pericardium. Initial imaging with echocardiography, cardiac magnetic resonance (CMR), and computed tomography (CT) revealed multilocular pericardial cysts. Surgical resection was performed under cardiopulmonary bypass (CPB), but complete excision was limited due to epicardial infiltration. Histopathology confirmed a benign mesothelial origin. One year later, recurrence prompted a second surgical intervention with total pericardiectomy and Gore-Tex patch reconstruction. Results: Postoperative recovery was uneventful in both instances. Follow-up imaging at 6 and 12 months demonstrated no significant recurrence. Histological analysis confirmed benign cysts lined with mesothelial cells, positive for calretinin and WT-1. This represents one of the first documented living cases of pericardial BMM managed with staged surgery and total pericardiectomy. Conclusions: Pericardial BMM is a rare, benign, but potentially recurrent lesion. In cases of extensive or recurrent disease, total pericardiectomy may offer definitive treatment. Multimodal imaging, histopathological evaluation, and personalized surgical planning are essential for effective management.

## 1. Introduction

Pericardial cystic diseases comprise a rare and diverse group of pathologies that include both congenital and acquired entities. These cysts may originate from developmental anomalies, infections, trauma, or neoplasms. Although pericardial cysts are most commonly benign and asymptomatic, their potential to mimic other conditions or cause hemodynamic compromise warrants careful evaluation and management. They are typically located in the right cardiophrenic angle and are incidentally discovered during imaging performed for unrelated reasons [1,2].

Primary pericardial tumors, including benign cysts and mesothelial proliferations, are extremely rare, with an estimated incidence of 1 in 100,000 [1]. Congenital pericardial cysts and diverticula arise from the failure of mesenchymal lacunae to coalesce during embryogenesis, whereas acquired lesions are often linked to prior inflammation, cardiac surgery, or metastatic disease [3].

Secondary involvement of the pericardium by metastases is more common than primary neoplasia, with melanoma being the most frequently reported cause of cardiac metastases. Over 50% of melanoma-related cardiac metastases involve the pericardium [3]. Among primary tumors, pericardial mesothelioma is a rare and aggressive tumor originating from mesothelial cells lining the pericardial sac [4]. A particularly rare subtype of benign mesothelial proliferation, termed benign multicystic mesothelioma (BMM), or multilocular mesothelial inclusion cysts, has been predominantly reported in the abdominal cavity and pelvis, with only a handful of documented cases affecting the pericardium [5,6,7,8,9].

We report a rare case of recurrent multilocular mesothelial inclusion cysts of the pericardium in a young woman with a prior history of malignant melanoma. The case highlights the diagnostic challenges, therapeutic considerations, and surgical strategies involved in managing this exceptionally rare entity, with a focus on personalized management and recurrence prevention.

## 2. Case Presentation

A 36-year-old woman presented with progressive exertional dyspnea, intermittent chest pain, and generalized fatigue. Her past medical history was significant for malignant melanoma, treated with wide excision and adjuvant therapy eight years prior. Routine surveillance had shown no evidence of recurrence.

**Figure 1:**
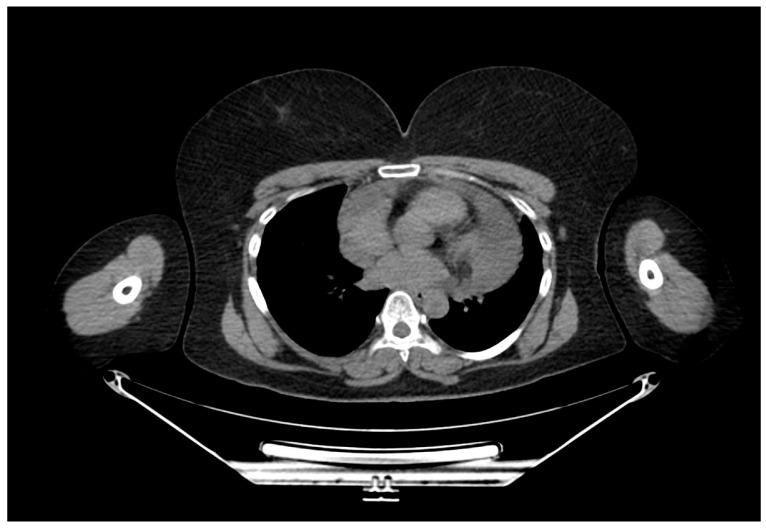
Axial view of the thoracic CT confirming massive pericardial effusion.

**Figure 2:**
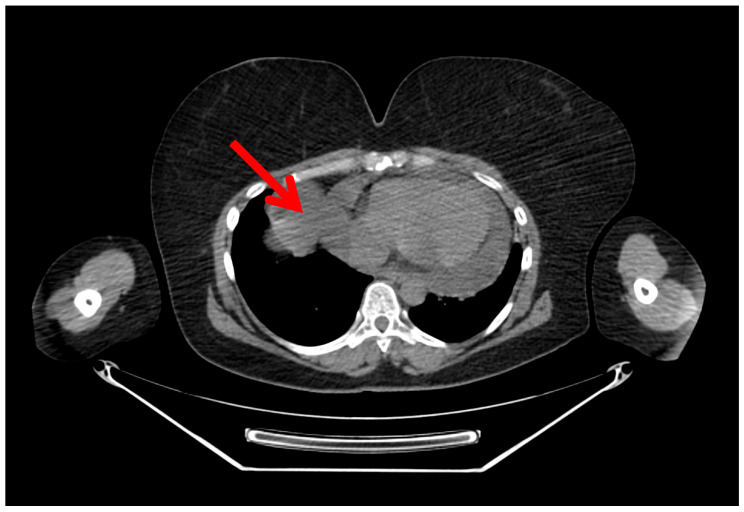
Axial view of the chest CT confirming fluid-filled sac-like connection to right pleural space (red arrow).

**Figure 3:**
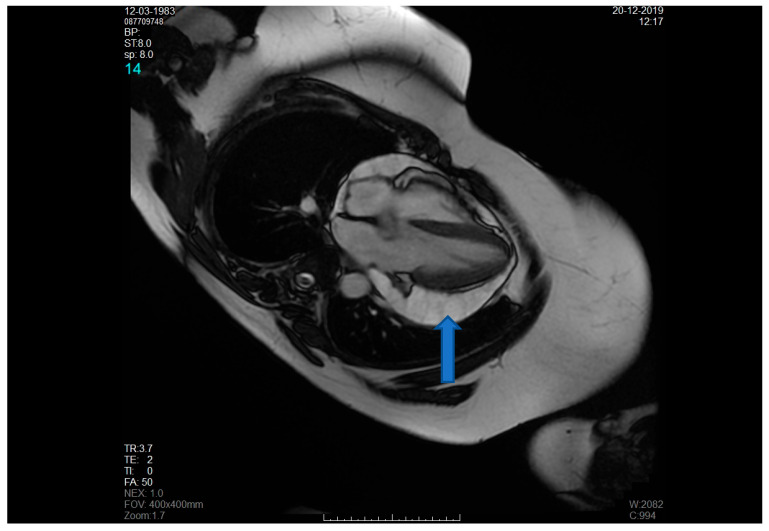
Cardiac MR showing cystic pericardial component (blue arrow) with no evidence of myocardial nodular infiltration.

Initial evaluation revealed muffled heart sounds on auscultation, without signs of cardiac tamponade. Transthoracic echocardiography (TTE) demonstrated a large pericardial effusion measuring approximately 2.5 to 3.0 cm surrounding both ventricles. Chest computed tomography (CT) confirmed a circumferential pericardial effusion, up to 30 mm in thickness, with a diverticulum extending toward the right pleura (Figure 1 and Figure 2). Cardiac magnetic resonance imaging (MRI) revealed a multilocular cystic formation, suggesting a pericardial origin without evidence of infiltration into myocardial tissue (Figure 3).

Given the risk of right ventricular injury and possible inefficiency, pericardiocentesis was deferred, and a surgical approach was pursued for both diagnostic and therapeutic purposes.

## 3. Initial Surgical Management

A subxiphoid approach was initially employed to access the pericardial space. Upon opening the pericardium, numerous translucent cysts of varying sizes were visualized, some of which infiltrated the epicardium and extended into the pleural cavity at the right cardio-phrenic angle. The anatomical complexity and the unknown etiology necessitated conversion to a full median sternotomy.

**Figure 4:**
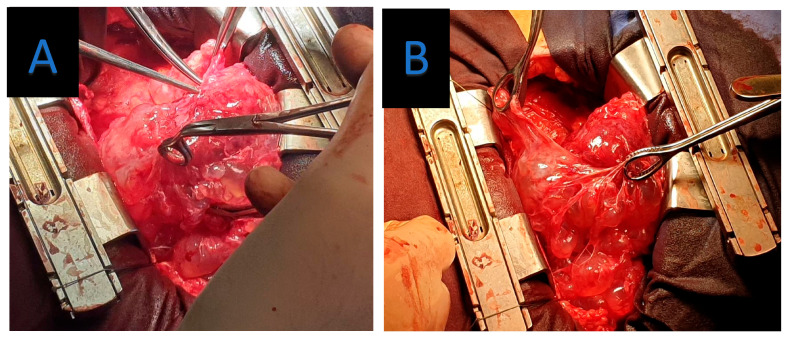
(**A**,**B**) Operative images demonstrating cystic extension of the pericardium.

**Figure 5:**
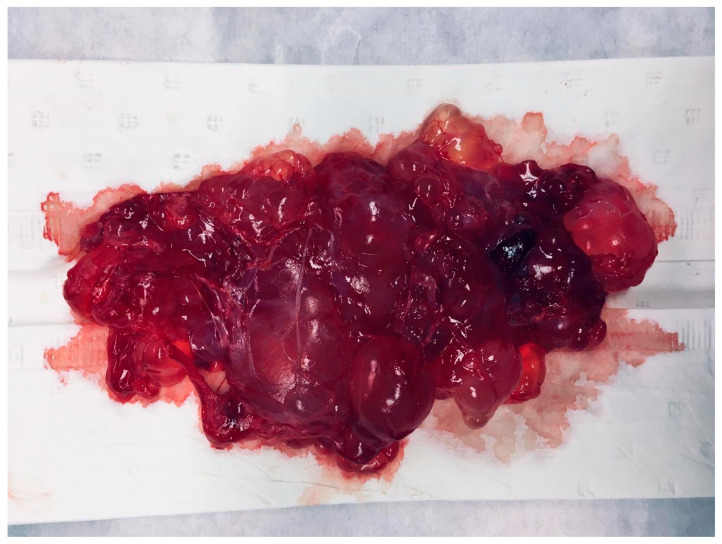
Gross image of the resected cystic cluster specimen.

To facilitate safe dissection and minimize myocardial manipulation, the surgery was performed under cardiopulmonary bypass (CPB). The pericardium was opened widely, revealing a multilocular cystic mass enveloping the epicardium and intrapericardial great vessels (Figure 4). Many cysts were successfully excised, with a close to complete resection despite their strong adherence to the epicardial surface (Figure 5). The decision was made to preserve the pericardium and remove all accessible cysts.

**Figure 6:**
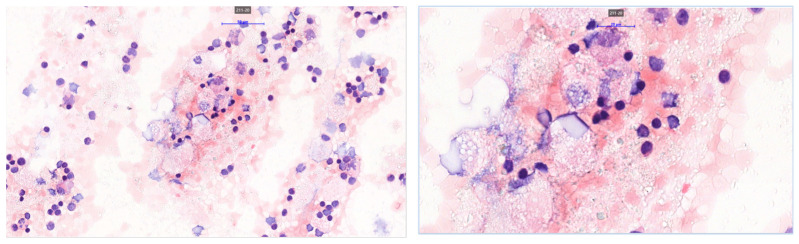
HE staining. 40× and 100× magnification from the cystic component. Cytospin preparation showing reactive macrophages and lymphoid cells. No malignant melanoma cells could be identified using special immunostains.

**Figure 7:**
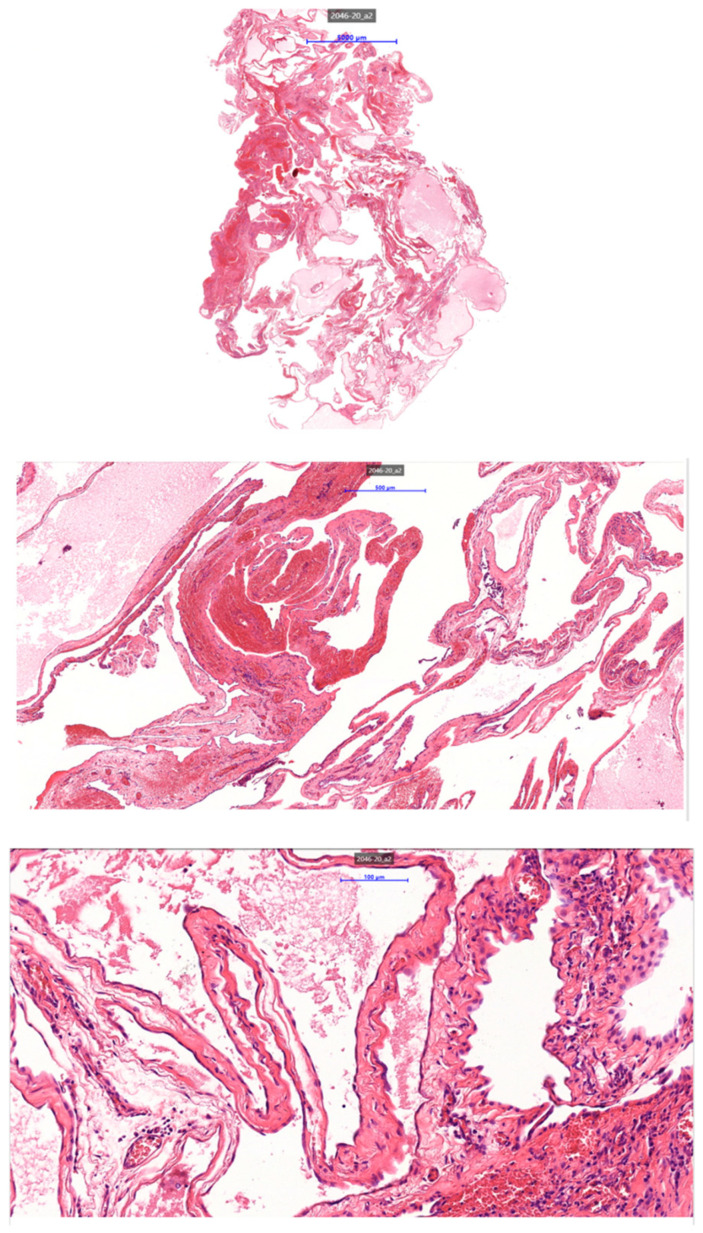
HE staining 10×, 20×, 40× magnifications. All of the pictures show a multilocular cyst with bland-looking mesothelium lining the cyst wall, indicative of a multilocular mesothelial inclusion cyst.

Histopathological analysis revealed cysts lined by a single layer of flattened to cuboidal mesothelial cells with fibrous stroma and no evidence of atypia or malignancy. Immunohistochemistry was positive for calretinin and WT-1, supporting a mesothelial origin (Figure 6 and Figure 7).

The patient’s postoperative course was uneventful, and she was discharged on the seventh postoperative day.

## 4. Recurrence and Second Operation

**Figure 8:**
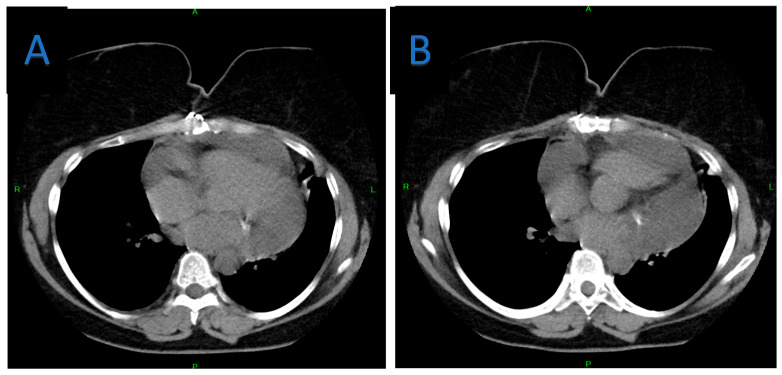
Axial chest CT images at one-year follow-up. (**A**,**B**) show evidence of pericardial mass, effusion.

At her one-year follow-up, routine TTE revealed recurrent pericardial fluid and cystic structures resembling those seen at the initial presentation. Chest CT confirmed a recurrence of multilocular cysts, again involving the epicardium and pericardial space (Figure 8).

**Figure 9:**
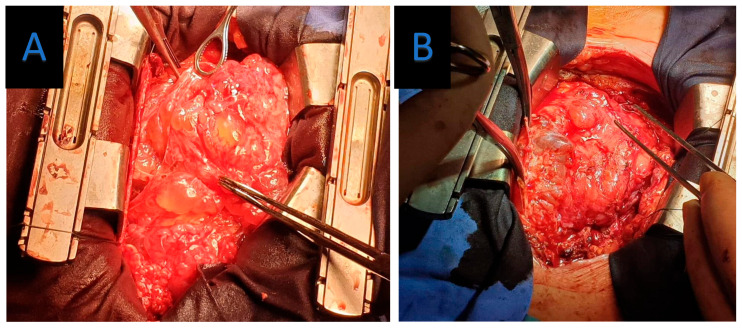
(**A**,**B**): Cystic adhesions to the epicardial surface observed during the second operation.

**Figure 10:**
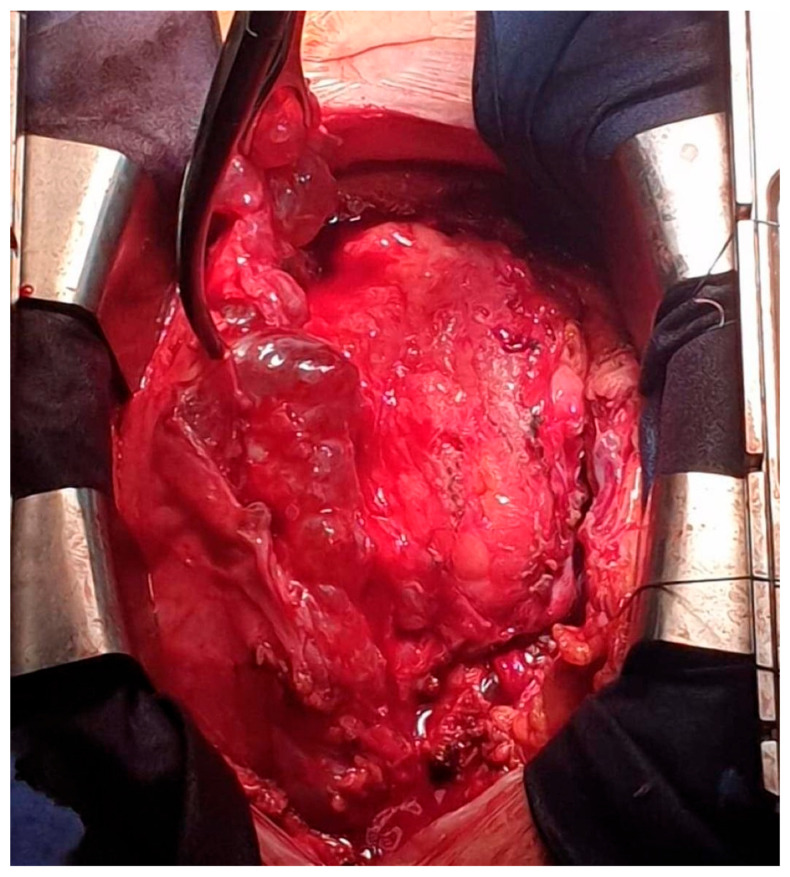
Complete cystic excision from the epicardium, the intraoperative image of the process.

Given the high likelihood of recurrence due to residual cystic tissue and the risk of complications, the patient underwent a second surgical intervention. After opening the pericardium, a multitude of cysts were noticed, which covered the epicardium around the heart (Figure 9). The cysts were completely removed (Figure 10), followed by a total pericardiectomy, involving resection of the pericardium from phrenic-to-phrenic nerve and removal of the diaphragmatic pericardial layer. The pericardial defect was reconstructed using a Gore-Tex prosthetic patch to facilitate further possible interventions with lower risk of myocardial injury at chest opening.

**Figure 11:**
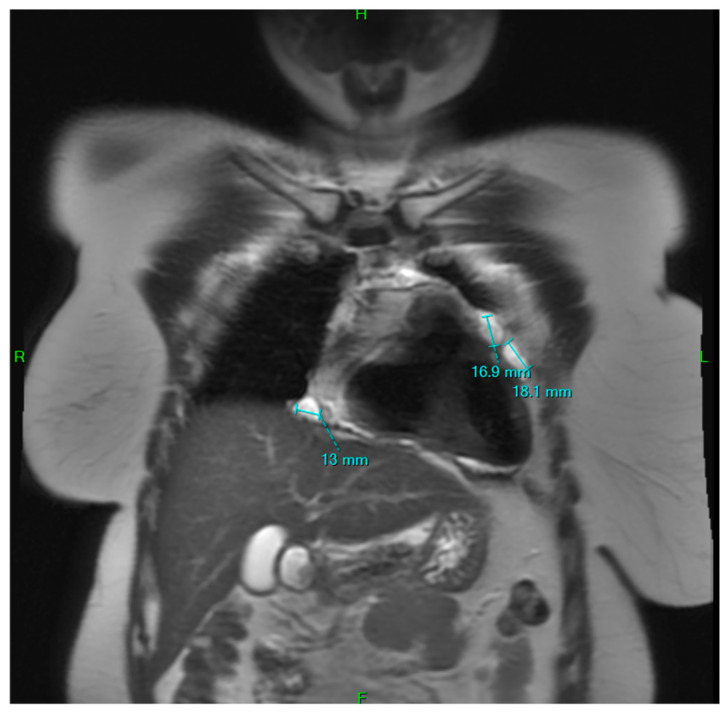
Follow-up cardiac MR at 4 years after the second intervention, coronal view, demonstrating subclinical recurrence.

**Figure 12:**
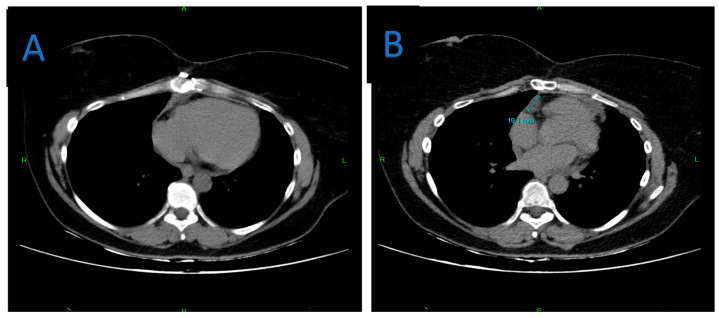
Follow-up chest CT, at 4 years after the second intervention axial view. (**A**) No evidence of pericardial effusion or other abnormalities at lower mediastinal level, (**B**) Subclinical recurrence demonstrated, only at the level of the heart and great vessels—at the junction of the upper and lower mediastinum.

The patient recovered well and was discharged on postoperative day 7. Follow-up imaging at 6–12 months and 4 years demonstrated no significant recurrence, with only small residual cysts near the subphrenic pericardium (which was impossible to resect) and were deemed clinically insignificant (Figure 11 and Figure 12).

## 5. Discussion

Pericardial masses—whether neoplastic, thrombotic, cystic, or congenital-like diverticula—are rare entities but can be significant contributors to morbidity and mortality. These lesions may be discovered incidentally in asymptomatic individuals during imaging performed for other reasons, or they may manifest with nonspecific symptoms such as chest discomfort, dyspnea, or palpitations. Transthoracic echocardiography is typically the first-line modality for detecting these masses, evaluating pericardial effusions, and identifying any hemodynamic impact. Nonetheless, advanced cross-sectional imaging techniques are often required to better define the nature of the mass and to assess its relationship with surrounding anatomical structures [5].

Benign multicystic mesothelioma is a rare entity that typically arises in the peritoneal cavity and affects young to middle-aged women. Thoracic BMM is even less frequent, with only a few cases of pericardial involvement reported in the literature, the majority diagnosed post-mortem [6,7,8,10]. There are other atypical localizations of mesothelial cysts intramyocardially, in the interventricular septum, or adherent to the external surface of the pericardium, compressing the right bronchus [9,11,12].

Other intrathoracic localizations include the mediastinal and pleural localizations. Primary mediastinal mesothelial cysts are uncommon; multilocular (“multicystic”) variants are even less frequent. A 2020 surgical case described a giant multilocular mesothelial cyst in the mediastinum, successfully resected thoracoscopically [13]. Such cases broaden the anatomic spectrum of BMM-like lesions beyond the peritoneum and emphasize the overlap of imaging features with bronchogenic, thymic, and Müllerian cysts—making histology and IHC decisive for diagnosis [14]. True pleural BMM is exceptionally rare, but cystic mesothelial proliferations of the pleura are documented. A 2022 report detailed a unilocular pleural mesothelial cyst completely removed by VATS with no recurrence at 2-year follow-up, illustrating that when confined to the pleura, these lesions are amenable to limited resection and tend to have favorable outcomes [15]. While pleural cases may be uni- rather than multicystic, they sit on the same benign mesothelial spectrum and enter the differential for cystic pleural masses [15]. Table 1 summarizes the clinical, diagnostic, management, and outcome specifics for the BMM with thoracic localization [14,15].

Histologically, BMM consists of cysts lined by mesothelial cells that may be flat, cuboidal, or columnar. These lesions are characterized by fibrous walls and absence of smooth muscle or malignant features. Immunohistochemical markers such as calretinin, cytokeratin, and WT-1 are used to confirm mesothelial origin [15].

Across thoracic sites, cysts are lined by flattened to cuboidal mesothelial cells that label for calretinin, WT1, D2-40 and broad cytokeratins (e.g., CK5/6, AE1/AE3); Müllerian (PAX8) and epithelial (CEA) markers are negative [13,16]. Current pathology guidance also notes that BAP1 loss is typical of malignant mesothelioma but is retained in benign lesions—helpful when morphology is subtle [16]. Differential diagnosis includes lymphangioma, thymic and bronchogenic cysts and well-differentiated papillary mesothelial tumor.

The etiology of BMM remains unclear. Some authors postulate a reactive process to prior inflammation or surgery, while others propose a neoplastic mechanism. Although BMM is considered benign, the risk of recurrence after incomplete resection is significant, with some reports indicating recurrence rates exceeding 40% [6,15]. Malignant transformation is exceedingly rare but has been documented in isolated cases.

These cysts are also known as mesothelial inclusion cysts, benign cystic mesotheliomas, or coelomic cysts. Their variable anatomical locations can be explained by embryologic development. During embryogenesis, the coelomic cavities—formed by the fusion of mesenchymal coelomic lacunae—give rise to the pleural and pericardial cavities on one side, and the peritoneal cavity on the other, separated by the developing septum transversum. A mesothelial cyst may form when fusion of a coelomic lacuna is incomplete, most commonly at the level of the pericardial coelom. Similar anomalies or secondary migratory processes can also occur at the level of the parietal pleura, mediastinal pleura, or septum transversum. Although the exact mechanism remains unclear, this embryologic pathway helps explain the occasional presence of mesothelial cysts in less common locations such as the chest wall, mediastinum, or diaphragm. Benign cystic mesothelioma more frequently develops in the abdomen, typically on the peritoneal surface, and is often linked to asbestos exposure [17].

The pathogenesis of benign multicystic mesothelioma (BMM) remains a topic of debate, with ongoing discussion as to whether it represents a reactive process or a true neoplasm. Proponents of a neoplastic origin highlight BMM’s tendency to recur even after surgical resection, which suggests an indolent but persistent growth pattern. In contrast, those who support a reactive etiology note its frequent association with prior abdominal surgeries or chronic inflammatory conditions such as pelvic inflammatory disease and endometriosis. Additionally, the marked predominance of BMM in women of reproductive age has led to speculation about a potential hormonal influence. However, efforts to confirm this through receptor studies have been largely inconclusive, with only a small number of cases showing estrogen or progesterone receptor positivity.

Genetic predisposition appears to be rare, with only a few reports suggesting a possible link. Some cases have described an association between BMM and Mediterranean Familial Fever, while others have documented familial clustering involving BMM and malignancies such as ovarian and colorectal cancer [18].

In terms of differential diagnosis, pericardial cysts may resemble other conditions on plain chest radiographs, such as pericardial fat pads or ventricular aneurysms. Diaphragmatic tumors, including teratomas, should also be considered, as they often contain both solid and cystic components. Lymphangiomas represent another possibility, typically appearing as multilocular or multicystic lesions. Additional differentials include Morgagni hernia and diaphragmatic eventration. Bronchogenic cysts, usually located in the middle mediastinum, may also be considered; however, they are characteristically lined with bronchial epithelium, a feature identifiable on histopathological examination [19].

Pericardial BMM might mimic clinical manifestations of other pericardial diseases resulting from various pathophysiological mechanisms, including inflammation (e.g., pericarditis), excess fluid accumulation (PEff), restriction of cardiac expansion due to pericardial stiffening (constrictive pericarditis, or CP), as well as the presence of masses (both benign and malignant), and congenital anomalies such as pericardial absence.

Pericarditis is generally classified based on its duration and underlying cause. Pericardial effusions are typically categorized by their volume, hemodynamic impact (presence or absence of cardiac tamponade physiology, or CTP), and fluid characteristics. CP is further divided into two key subtypes:

- Transient constrictive pericarditis (TCP): where constriction resolves spontaneously or with anti-inflammatory treatment over time.
- Effusive-constrictive pericarditis (ECP): where constriction becomes evident after the initial presentation, typically following removal of the effusion.

The clinical presentation of pericardial disorders ranges from chest pain and signs of systemic inflammation to hemodynamic instability or chronic heart failure. Symptoms may be dramatic, as in acute pericarditis, or subtle, especially when occurring alongside systemic disease. Pericardial effusion may be entirely asymptomatic and discovered incidentally. Additionally, pericardial disease can provoke arrhythmias such as sinus tachycardia, atrial arrhythmias, and notably, atrial fibrillation.

In response to injury, the pericardium undergoes several changes: desquamation of mesothelial cells, increased vascular permeability, neovascularization, and exudation of fluid, fibrin, and inflammatory cells. During the repair phase, granulation tissue forms with proliferating fibroblasts and fragile new blood vessels. This process can result in focal or diffuse adhesions between the parietal and visceral layers of the pericardium and can lead to pericardial thickening, ultimately contributing to the development of CP [20].

Although more than half of mesothelial cysts are clinically silent, their potential to compress adjacent structures—including the heart, great vessels, and lungs—can result in serious complications such as respiratory failure, arrhythmias, and cardiac arrest. Additional risks include infection, hemorrhage, and rupture. In cases where surgical intervention is contraindicated, percutaneous drainage has been shown to provide symptomatic relief. However, to prevent recurrence and mitigate the risk of secondary complications such as infection, complete surgical excision remains the definitive treatment of choice [14].

Differentiation from other pericardial masses is essential, as BMM may mimic malignant mesothelioma, metastatic disease, or congenital cysts [21]. Therefore, as demonstrated in our case presentation, multimodal imaging, including echocardiography, CT, and MRI, plays an important role in preoperative assessment. However, in our opinion, definitive diagnosis obviously relies on histopathological evaluation and immunohistochemistry, which is a protocol requirement for any abnormal tissue resected surgically.

This case is notable for several reasons:

- It represents the first living report of pericardial BMM managed with staged surgery.
- The lesion’s multilocular and highly adherent pattern to the epicardium required CPB-assisted dissection.
- Complete pericardiectomy was essential in achieving long-term disease control.

Given the benign nature of the lesion, complete surgical excision remains the treatment of choice. When cysts are adherent to both the epicardium and pericardium, lack of pericardial excision may lead to recurrence, as seen in our case. Therefore, a strategy of total pericardiectomy, combined with resection of involved cysts and prosthetic pericardial reconstruction, may be considered curative in patients with extensive disease.

Both the 6-month, 1- and 4-year follow-up chest CT scan and MRI showed only subclinical recurrence at the level of the subphrenic pericardium (Figure 11 and Figure 12), which posed too much risk to be resected, due to the proximity of the phrenic nerve. However, this also clearly emphasizes the necessity of extensive pericardiectomy to minimize recurrence.

Therefore, long-term surveillance with echocardiography and cross-sectional imaging is essential due to the risk of late recurrence [5]. Personalized treatment strategies, tailored to the extent of disease and patient-specific risk factors, are necessary to optimize outcomes.

Furthermore, a possible hereditary etiology could be explored through genetic screening, as a future diagnostic tool.

## 6. Conclusions

Multilocular mesothelial inclusion cysts of the pericardium are exceedingly rare and may recur following incomplete surgical excision. We present a unique case of recurrent pericardial BMM managed with staged resection and complete pericardiectomy. This case underscores the importance of multimodal imaging, histological confirmation, and individualized surgical planning. Based on this comprehensive case presentation, total pericardiectomy may provide definitive treatment and also prevent recurrence.

## Figures and Tables

**Table 1:** Benign Multicystic Mesothelioma in the Chest: Pericardial/Cardiac, Mediastinal, and Pleural Sites.

Site	Typical Patient Presentation	Imaging Hallmarks	Key Histopathology/IHC	Management	Outcomes Reported	Differential Diagnosis and Practical Notes
Pericardium/cardiac	Young to middle-aged adults; often incidental discovery or chest discomfort	Well-circumscribed, multiloculated cystic pericardial mass; can mimic congenital pericardial cyst on CT/MRI	Thin-walled multicysts lined by bland mesothelial cells; calretinin+, WT1+, D2-40+, CK5/6+; PAX8−; BAP1 retained	Complete surgical excision (VATS)	Uneventful recovery; no recurrence at ~18 months	Low threshold for tissue diagnosis when a “pericardial cyst” is large, septated, or enhancing; confirm mesothelial lineage with IHC; exclude Müllerian cyst and lymphangioma.
Mediastinum	Adults; dyspnea/chest fullness or incidental mass	Giant, multiloculated cystic lesion (often anterior mediastinum) on CT	Mesothelial lining with bland cytology; calretinin+, WT1+, D2-40+, broad cytokeratins; BAP1 retained	Thoracoscopic complete resection	No recurrence at 2 years	Imaging overlaps with bronchogenic and thymic cysts, Müllerian cysts, and lymphangioma—histology and IHC decisive.
Pleura	Middle-aged adults; cough or incidental finding	Unilocular or pauci-septate pleural cystic lesion; may abut mediastinal pleura	Mesothelial cyst wall; calretinin+, WT1+, CK5/6+; PAX8−; BAP1 retained	VATS excision (wedge/cystectomy)	Disease-free at 24 months	Ensure exclusion of lymphangioma and Müllerian cyst; true multicystic pleural lesions are exceptional.
Cross-site diagnostic & reporting guidance		Radiology–pathology correlation emphasized for cystic mesothelial lesions across pleura/pericardium/mediastinum	Use mesothelial panel (calretinin, WT1, D2-40, CK5/6) and exclusion markers (e.g., PAX8, CEA). BAP1/MTAP loss suggests malignancy; retention supports benignity	—	—	Apply 2023 IMIG consensus recommendations for distinguishing benign vs. malignant mesothelial proliferations
Follow-up recommendations	All patients post-complete resection	Cross-sectional imaging (CT or MRI) at 6–12 months then annually for at least 2–3 years	Histology and IHC review if any suspicious recurrence noted	Surveillance tailored to site and completeness of resection	No recurrences reported in the literature up to 24 months	Ensure follow-up to detect rare recurrences and exclude malignant transformation.

## Data Availability

No new data were created or analyzed in this study. Data sharing is not applicable to this article.

## Abbreviations

BMM	Benign multicystic mesothelioma
TTE	Transthoracic echocardiography
CT	Computed tomography
MRI	Magnetic resonance imaging
CPB	Cardiopulmonary bypass
IHC	Immunohistochemistry
VATS	Video-Assisted Thoracoscopic Surgery
CEA	Carcinoembryonic Antigen
PEff	Excess fluid accumulation
CP	Constrictive Pericarditis
CTP	Cardiac tamponade physiology
TCP	Transient constrictive pericarditis
ECP	Effusive-constrictive pericarditis
